# Supplementary material for: Immunization with V987H-stabilized Spike glycoprotein protects K18-hACE2 mice and golden Syrian hamsters upon SARS-CoV-2 infection
Source: Nat Commun. 2024 Mar 21;15:2349. doi: 10.1038/s41467-024-46714-w (PMC10957958; doi:10.1038/s41467-024-46714-w)
Supplement: Supplementary file 3 — Supplementary Information [file 41467_2024_46714_MOESM3_ESM.pdf]

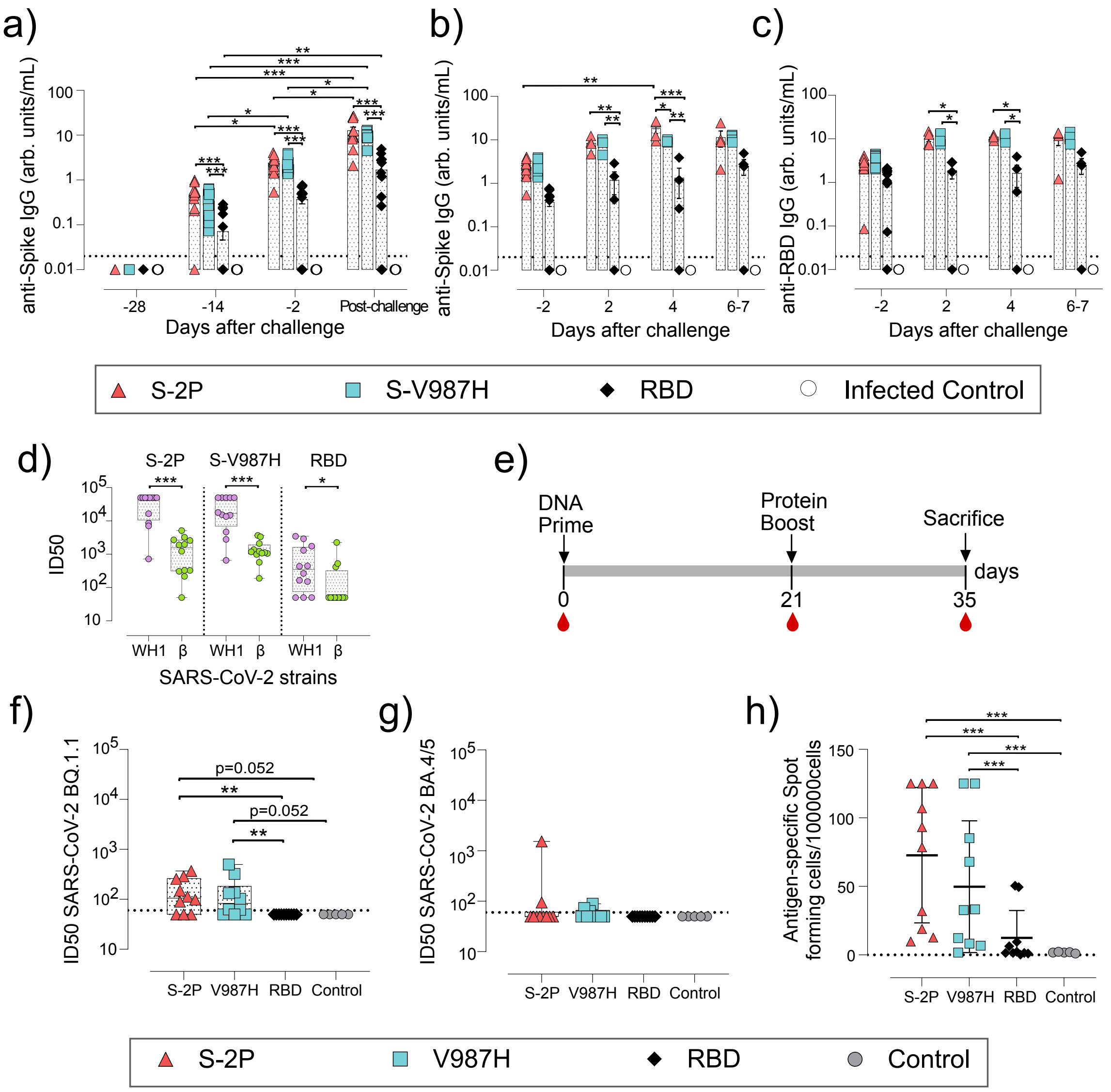


**Supplementary Fig. 1. Analysis of humoral and cellular responses in C57Bl/6 mice and the antibody response in immunized K18-hACE2 mice challenged with SARS-CoV-2 D614G.** **a** Kinetics of anti-S IgG antibodies in serum samples of immunized and challenged K18-hACE2 mice expressed as arbitrary units (arb. units) per mL. Red triangles: S-2P group (n= 16). Blue squares: S-V987H group (n= 14). Black diamonds: RBD (n=15). White circles: unvaccinated and challenged mice (infected control) (n=19). Groups in each time point were analyzed using two-sided Conover-Iman test with multiple comparison correction by FDR. Differences among animals within a particular group along time were analyzed using two-sided Friedman test corrected for multiple comparison using FDR. SARS-CoV-2 D614G challenge was performed on day 0. **b** and **c** Kinetics of anti-S and anti-RBD IgG antibodies respectively, in serum samples from K18-hACE2 mice on days -2, 2, 4 and 6-7 (endpoint) after SARS-CoV-2 D614G challenge. S-2P n=16 on day -2, n=4 on days 2, 4 and 6-7. S-V987H n=14 on day -2, n=4 on day 2, 4, and 6-7. RBD n=15 on day -2, n=4 on days 2, 4 and 6-7. Infected control n=19 on day -2, n=4 on day 2 and 4, n=8 on days 6-7. Mean plus standard error of the mean (SEM) is shown. Statistical analyzed was performed as in **a**. **d** Neutralizing activity against SARS-CoV-2 WH-1 (purple) and B.1.351 (Beta) (green) variants of serum samples from immunized K18-hACE2 mice after viral challenge. S-2P n= 16; S-V987H n= 14; and RBD n=15 Differences among neutralizing activity were analyzed using two-sided Mann-Whitney test. **e** C57Bl/6 immunization schedule. Mice were immunized firstly with DNA and boosted with recombinant protein adjuvanted with AdjuPhos. Two weeks after booster, serum samples and splenocytes were collected. **f** and **g** Neutralizing activity of serum samples from immunized C57BL/6 mice (n=10 per immunization group, control group n=5) against SARS-CoV-2 Omicron BQ1.1 and BA4/5, respectively. **h** Spike-specific T

cell responses determined as IFN-γ producing splenocytes from immunized C57BL/6 (n=10 per immunized group, control group n=5). Data are shown as number of positive spots/100000 cells. Differences between groups for neutralizing titers and cellular responses were analyzed using two-sided Peto & Peto left-censored k sample test corrected by FDR. * $p<0.05$, ** $p<0.01$, *** $p<0.001$. P values proximal to statistical significance are shown as numbers. Mean plus standard error of the mean (SEM) is shown. Source data are provided as a Source Data Supplementary Figure 1.

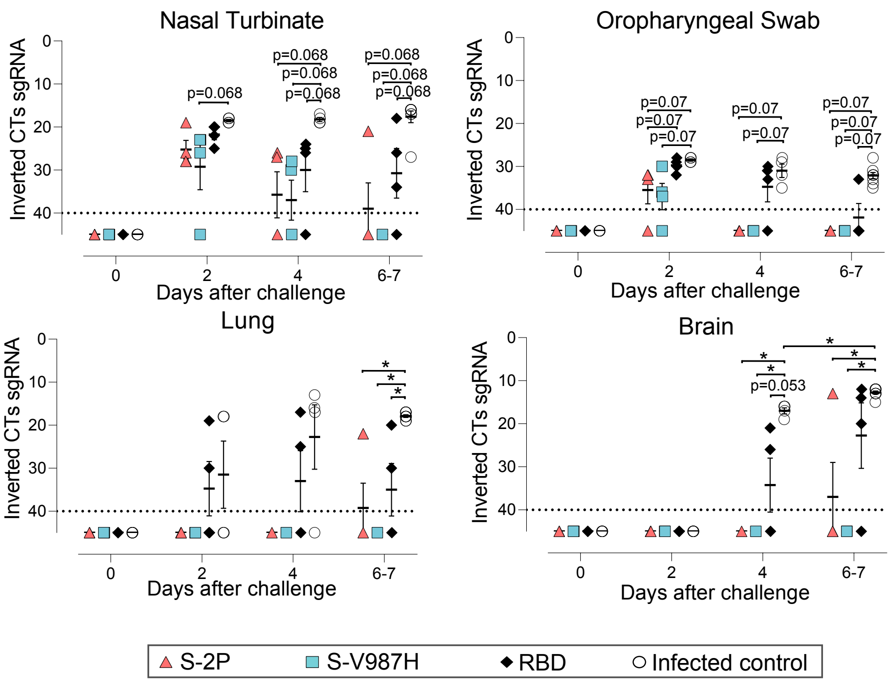


**Supplementary Fig. 2. Analysis of tissue sub-genomic RNA in immunized K18-hACE2 mice challenged with SARS-CoV-2 D614G.** Levels of SARS-CoV-2 subgenomic RNA (represented as inverted Ct) in oropharyngeal swabs, nasal turbinate, lung, and brain after virus challenge. Four mice per timepoint were analyzed, with two exceptions: 1) day 0: S-V987H n=2, RBD n=3, and infected control n=3; and 2) days 6-7: infected control n=8. Dot line indicates limit of detection (Ct=40). Differences between groups were analyzed using two-sided Peto & Peto left-censored k sample test corrected by FDR. * $p<0.05$. P values proximal to statistical significance are shown as numbers. Mean plus standard error of the mean (SEM) is shown. Source data are provided as a Source Data Supplementary Figure 2.

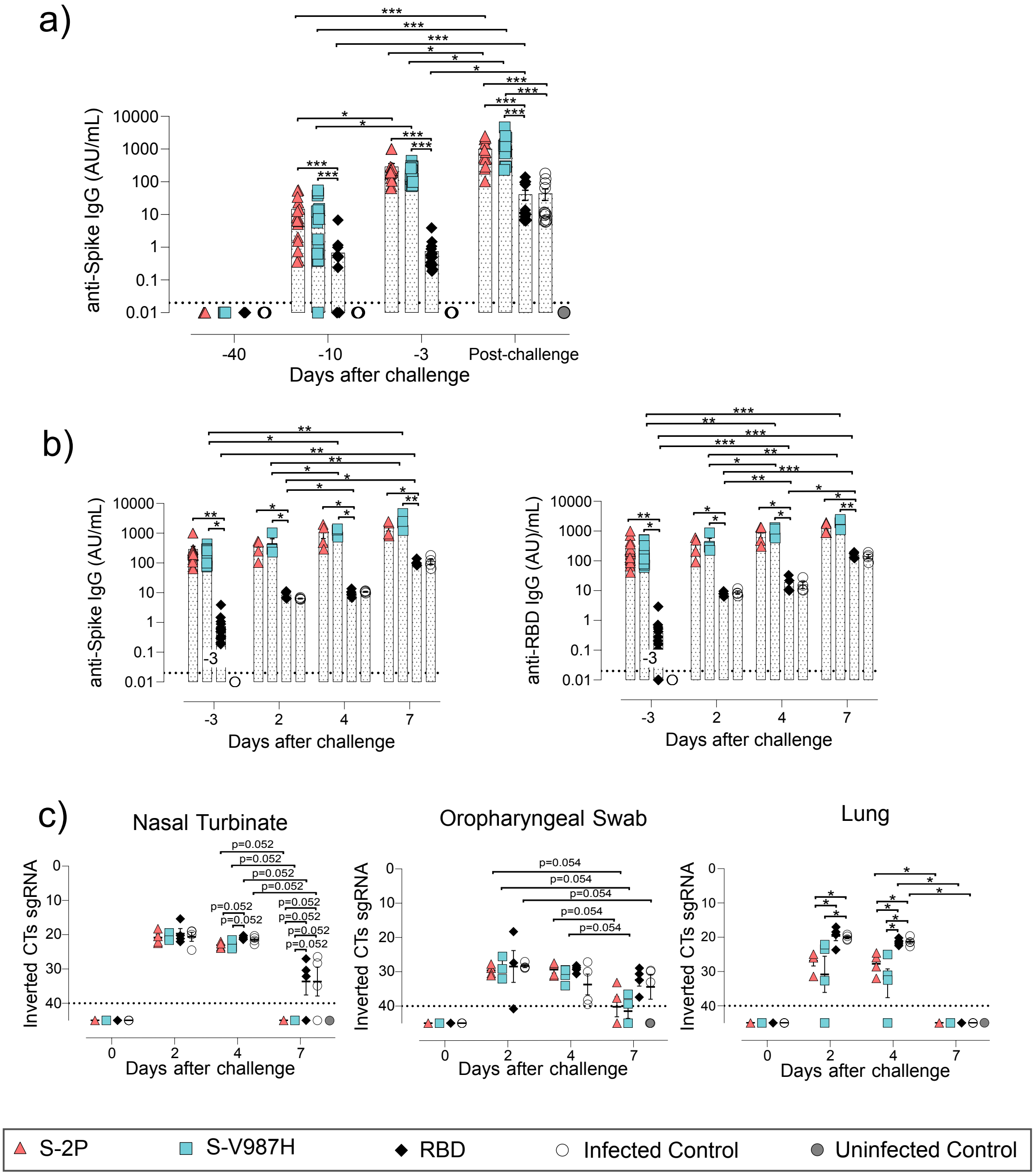


**Supplementary Fig. 3. Analysis of anti-S IgG responses and levels of tissue sub-genomic RNA in immunized golden Syrian hamsters challenged with SARS-CoV-2 D614G.**

**a** Kinetics of anti-S antibodies in serum samples expressed as arbitrary units (arb. units). Red triangles: S-2P group (n= 16). Blue squares: S-V987H group (n= 16). Black diamonds: RBD (n=16). White circles: unvaccinated and infected GSH (n=16). Grey circles: unvaccinated and unchallenged GSH (n=4). Groups in each time point were analyzed using two-sided Conover-Iman test with multiple comparison correction by FDR. Differences among animals within a particular group along time were analyzed using two-sided Friedman test corrected for multiple comparison using FDR. **b** Kinetics of anti-Spike and anti-RBD IgG antibodies in serum samples on days -3, 2, 4 and 7 after SARS-CoV-2 D614G challenge. Mean plus standard error of the mean (SEM) are shown. N=16 on day -3, n=4 on day 2,4 and 7 for all three challenged groups. Differences were analyzed as indicated in **a**. **c** Levels of SARS-CoV-2 subgenomic RNA (represented as inverted Ct) in oropharyngeal swabs, nasal turbinate, and lung after virus challenge (n=4

per group and timepoint). Dot line indicates limit of detection (40 Cts). Mean plus standard error of the mean (SEM) are shown. Differences between groups were analyzed using two-sided Peto & Peto left-censored k sample test corrected by FDR. * $p<0.05$, ** $p<0.01$, *** $p<0.001$. P values proximal to statistical significance are shown as numbers. Source data are provided as a Source Data Supplementary Figure 3.

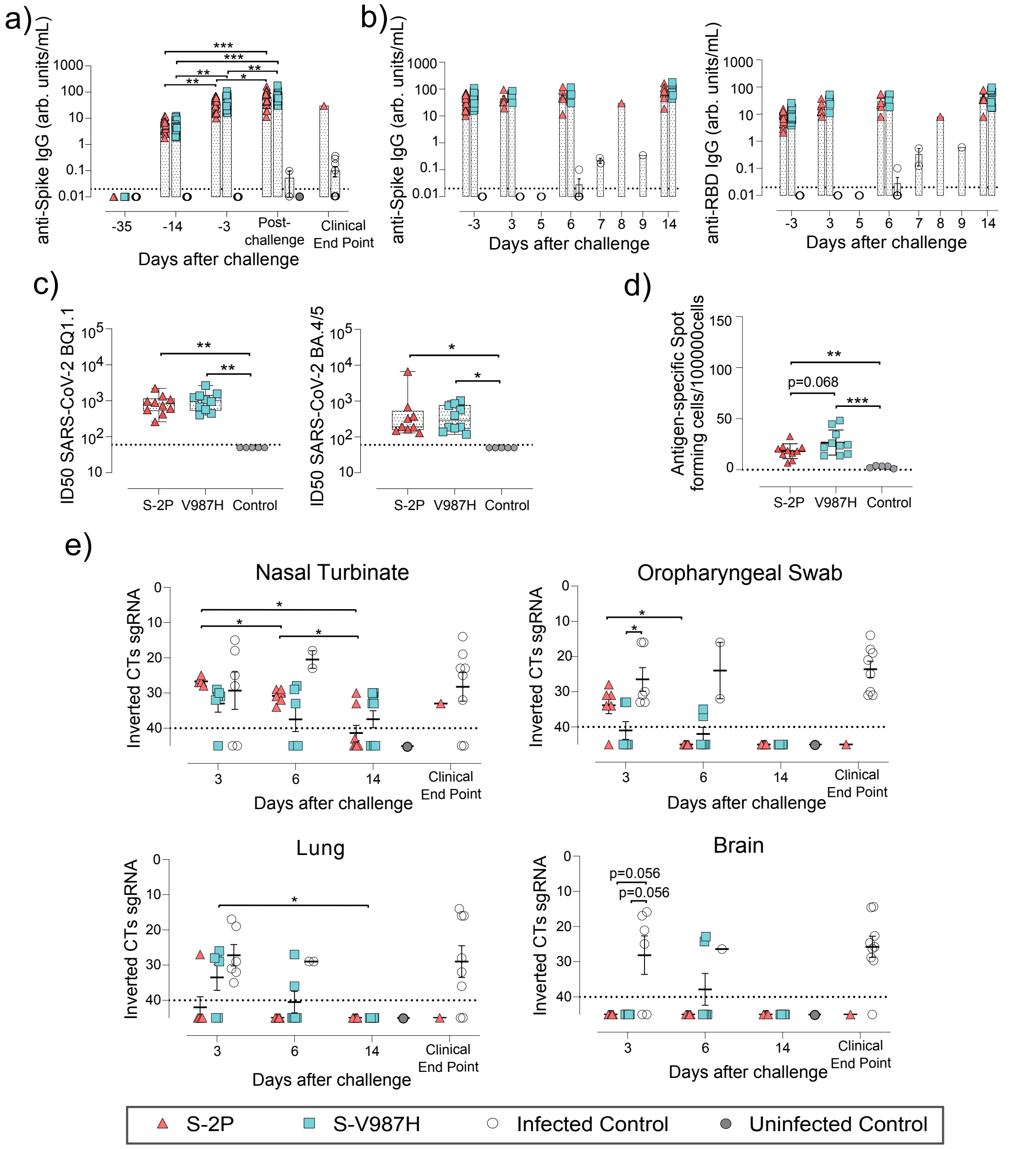


**Supplementary Fig. 4. Analysis of humoral and T cell responses in immunized C57BL/6 mice, and anti-S IgG responses and levels of tissue sub-genomic RNA in immunized K18-hACE2 mice challenged with SARS-CoV-2 B.1.351 (Beta) variant.** **a** Kinetics of anti-S IgG antibodies in serum samples from immunized K18-hACE2 mice challenged with the SARS-CoV-2 B.1.351 (Beta) variant expressed as arbitrary units (arb. units). Red triangles: S-2P group (n= 21 on days -35, -14 and -3; n=20 post-challenge, n=1 clinical end point). Blue squares: S-V987H group (n= 21 on all timepoints). White circles: unvaccinated-challenged mice (Infected control) (n=16 on days -35, -14 and -3; n=8 post-challenge, and n=8 clinical endpoints). Grey circles: uninfected and unvaccinated mice (n=10) (Uninfected control). Groups in each time point were analyzed using two-sided Conover-Iman test with multiple comparison correction by FDR. Differences among animals within a particular group along time were analyzed using two-sided Friedman test corrected for multiple comparison using FDR. **b** Kinetics of anti-S and anti-RBD IgG antibodies in serum samples from immunized K18-hACE2 mice on days -3, 2, 4, 6-9 (end point) and 14 after SARS-CoV-2 B.1.351 challenge. Data are

expressed as arbitrary units (arb. units). S-2P group: n=21 on day -3, n=6 on day 3, n=6 on day 6, n=1 on day 8 (clinical endpoints), and n= 8 on day 14. S-V987H group: n=21 on day -3, n=6 on day 3, n=6 on day 6, n= 9 on day 14. Unvaccinated-challenged mice (Control infected): n=16 on day -3, n=6 on day 3, n=2 on day 6, n= 8 on clinical endpoints. Uninfected and unvaccinated mice: n=10 on day 14. Statistical analysis was performed as indicated in **a**. Mean plus standard error of the mean (SEM) is shown. **c** Neutralizing activity of serum samples from immunized C57BL/6 mice (n=10 per immunization group, n=5 control group) against SARS-CoV-2 Omicron BQ1.1 and BA4/5. C57Bl/6 mice were immunized twice with recombinant protein (three weeks between doses). AddaVax was used as adjuvant. Two weeks after booster, serum samples and splenocytes were collected. **d** Spike-specific T cell responses determined as IFN-γ producing splenocytes from immunized C57BL/6 (n=10 in S-2P and S-V987H groups, and n=5 in the control group). Data are shown as number of positive spots/100,000 cells. **e** Levels of SARS-CoV-2 subgenomic RNA (represented as inverted Ct) in oropharyngeal swabs, nasal turbinate, lung, and brain after virus challenge. S2P: n=6 on day 3 and 6, n=1 on day 8 (clinical endpoint) and n=8 on day 14 after challenge. S-V987H group: n=6 on day 3 and 6, n=9 on day 14. Unvaccinated-challenged mice (Infected control): n=6 on day 3, n=2 on day 6, n= 8 on clinical endpoints. Uninfected and unvaccinated mice (Uninfected control): n=10 on day 14. Dot line indicates limit of detection (40 Cts). Differences between groups in **c**, **d** and **e** were analyzed using two-sided Peto & Peto left-censored k sample test corrected by FDR. * $p<0.05$, ** $p<0.01$, *** $p<0.001$. Source data are provided as a Source Data Supplementary Figure 4.

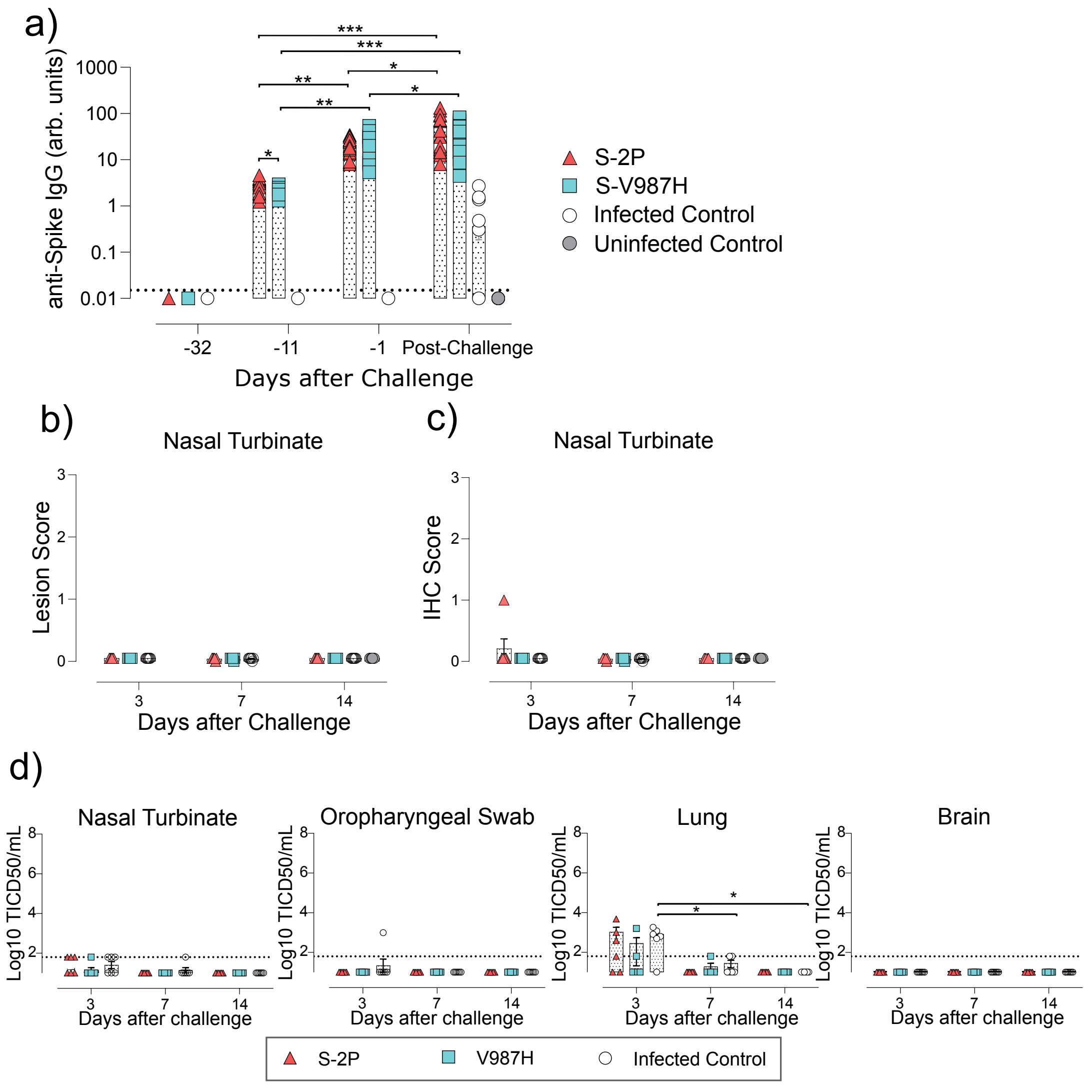


**Supplementary Fig. 5. Analysis of anti-Spike IgG responses, presence of NP and damage in nasal turbinate, and levels of infectious virions in tissues of immunized K18-hACE2 mice challenged with SARS-CoV-2 Omicron BQ1.1.**

**a** Kinetics of anti-S IgG antibodies in serum samples expressed as arbitrary units (arb. units). Red triangles: S-2P group (n= 18). Blue squares: S-V987H group (n= 18). White circles: unvaccinated/challenged mice (n=18) (Infected control). Gray circles unvaccinated/ no challenged mice (n=6) (Uninfected control). Groups in each time point were analyzed using two-sided Conover-Iman test with multiple comparison correction by FDR. Differences among animals within a particular group along time were analyzed using two-sided Friedman test corrected for multiple comparison using FDR. **b** Histopathological analysis of nasal turbinate by hematoxylin and eosin staining (n=6 per group and per timepoint after challenge). Lesion score: (0) no, (1) mild, (2) moderate, and (3) severe lesion. **c** Detection of SARS-CoV-2 nucleocapsid protein in nasal turbinate by immunohistochemistry (n=6 per group and timepoint after challenge). Staining score: (0) no, (1) low, (2) moderate, and (3) high amount of viral antigen. Differences between groups in **b** and **c** were analyzed using two-sided Asymptotic Generalized Pearson Chi-Squared test with FDR correction. **d** Titer of infectious virions determined in samples from nasal turbinate, oropharyngeal swab, lung and brain (n=6 per group and timepoint). Data are shown as Log10 of Median Tissue Culture Inhibition Dose per mL (TCID50/mL) and analyzed using two-sided Peto & Peto left-censored k sample test corrected by FDR. Statistically significant differences are indicated as follows: * p<0.05, ** p<0.01, *** p<0.001. P values proximal to statistical significance are shown as numbers. Mean plus standard error of the mean (SEM) is shown. Source data are provided as a Source Data Supplementary Figure 5.

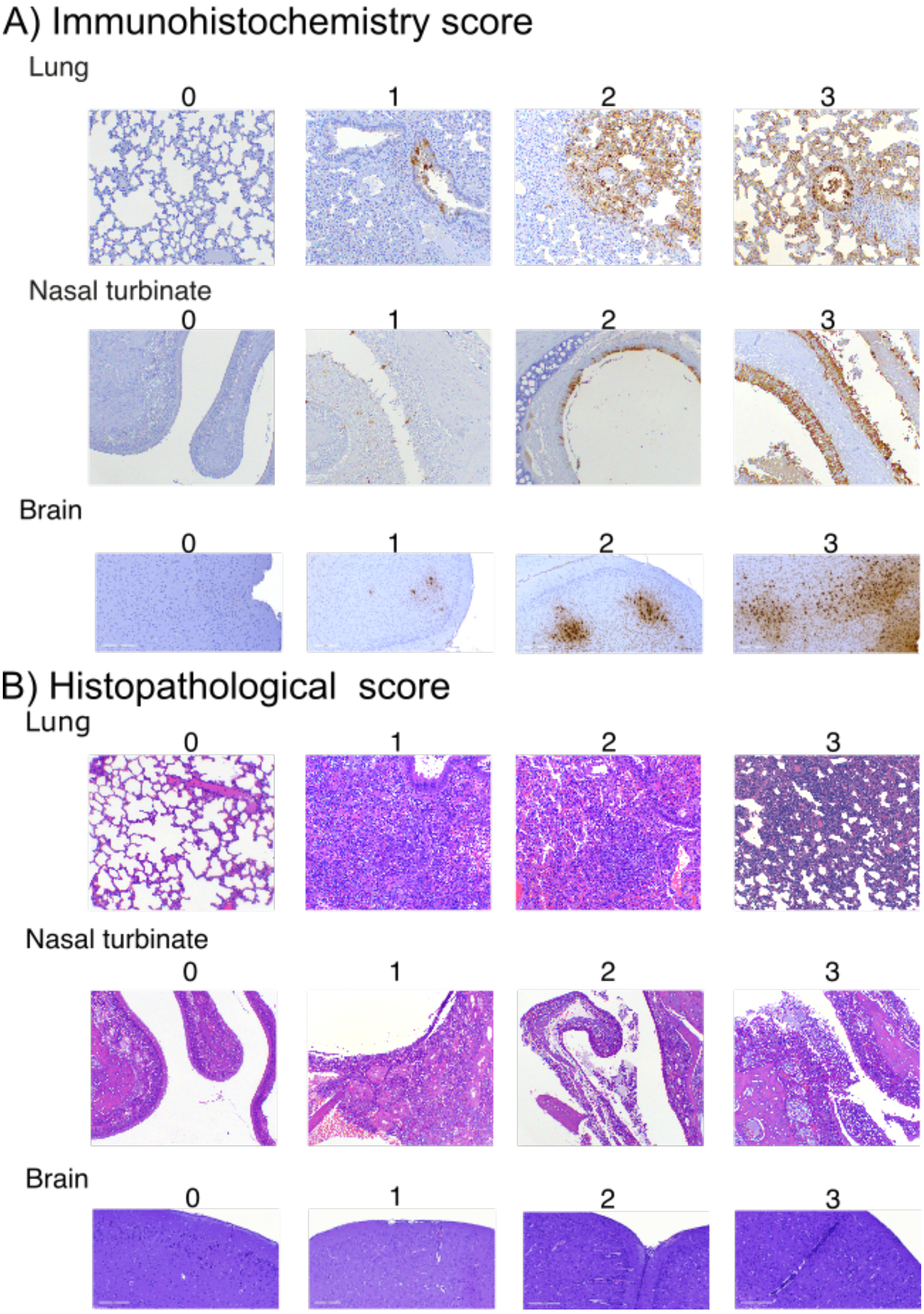


**Supplementary Fig. 6. Immunohistochemistry and Histopathological scores.**
